# Naturally acquired antibodies against *Plasmodium vivax* pre-erythrocytic stage vaccine antigens inhibit sporozoite invasion of human hepatocytes in vitro

**DOI:** 10.1038/s41598-024-51820-2

**Published:** 2024-01-13

**Authors:** Francis Babila Ntumngia, Surendra Kumar Kolli, Pradeep Annamalai Subramani, Samantha J. Barnes, Justin Nicholas, Madison M. Ogbondah, Brian B. Barnes, Nichole D. Salinas, Pongsakorn Thawornpan, Niraj H. Tolia, Patchanee Chootong, John H. Adams

**Affiliations:** 1 Center for Global Health and Interdisciplinary Research, University of South Florida, Tampa, FL USA; 2 Department of Molecular Medicine, Morsani College of Medicine, University of South Florida, Tampa, FL USA; 3 College of Marine Science, University of South Florida, St Petersburg, FL USA; 4 Host Pathogen Interactions and Structural Vaccinology Section, Laboratory of Malaria Immunology and Vaccinology, National Institute of Allergy and Infectious Diseases, National Institutes of Health, Bethesda, MD USA; 5 Department of Clinical Microbiology and Applied Technology, Faculty of Medical Technology, Mahidol University, Bangkok, Thailand

**Keywords:** Vaccines, Protein vaccines, Malaria

## Abstract

In *Plasmodium vivax*, the most studied vaccine antigens are aimed at blocking merozoite invasion of erythrocytes and disease development. Very few studies have evaluated pre-erythrocytic (PE) stage antigens. The *P. vivax* circumsporozoite protein (CSP), is considered the leading PE vaccine candidate, but immunity to CSP is short-lived and variant specific. Thus, there is a need to identify other potential candidates to partner with CSP in a multivalent vaccine to protect against infection and disease. We hypothesize that sporozoite antigens important for host cell infection are considered potential targets. In this study, we evaluated the magnitude and quality of naturally acquired antibody responses to four *P. vivax *PE antigens: sporozoite surface protein 3 (SSP3), sporozoite protein essential for traversal 1 (SPECT1), cell traversal protein of ookinetes and sporozoites (CelTOS) and CSP in plasma of *P. vivax* infected patients from Thailand. Naturally acquired antibodies to these antigens were prevalent in the study subjects, but with significant differences in magnitude of IgG antibody responses. About 80% of study participants had antibodies to all four antigens and only 2% did not have antibodies to any of the antigens. Most importantly, these antibodies inhibited sporozoite infection of hepatocytes in vitro. Significant variations in magnitude of antigen-specific inhibitory antibody responses were observed with individual samples. The highest inhibitory responses were observed with anti-CelTOS antibodies, followed by anti-SPECT1, SSP3 and CSP antibodies respectively. These data highlight the vaccine potential of these antigens in protecting against hepatocyte infection and the need for a multi-valent pre-erythrocytic vaccine to prevent liver stage development of *P. vivax* sporozoites.

## Introduction

*Plasmodium vivax* malaria is a global health problem in many tropical and sub-tropical countries of the world, with > 70% of cases occurring in Asia and the Americas^1–3^. An effective vaccine that provides protection and prevents transmission is considered the most cost-effective tool for malaria control and would greatly facilitate *P. vivax* elimination. The best targets for malaria vaccine development are parasite antigens that can induce an effective immune response in natural and experimental infections that is capable of inhibiting host cell invasion and parasite development^4,5^. To this respect, promising candidates are parasite proteins that play an important role in targeting cell infection.

Research into *P. vivax* vaccine targets have focused mainly on blood stage antigens, with very few studies on pre-erythrocytic (PE) antigens. Sporozoites, the infective stage of the malarial parasite are considered ideal targets for antimalarial strategies and protective immunity^6^. Sporozoites constitute a bottleneck in the parasite complex life cycle as only a very few sporozoites are injected by an infected mosquito^7^, they have a longer exposure time to the host immune system than antigens of blood-stage invasive stages^8^, and the PE stages are clinically silent. Liver infection is an obligatory step in malarial transmission. Once injected into the skin, sporozoites actively migrate in the dermis, traverse the capillary epithelium into the bloodstream and through the liver sinusoids into the parenchyma where they invade host hepatocytes, proliferate, and develop into exoerythrocytic forms (EEFs) inside a parasitophorous vacuole. Thus, PE vaccines are aimed at targeting the sporozoites and the EEFs, thereby preventing progression of the parasite to the blood stage. In other *Plasmodium* spp., sporozoite antigens are considered good vaccine candidates as antibodies against these antigens represent the first line of defense against infection. Studies have demonstrated that subunit vaccines based on sporozoite surface antigens and attenuated whole sporozoites can induce protection in both animal models and humans^9^. Orthologues of sporozoite antigens from other *Plasmodium* spp. are also present in *P. vivax* and have been shown to play critical roles during hepatocyte infection. Amongst them, the *P. vivax* circumsporozoite surface protein (CSP), the dominant molecule on the sporozoite surface is a leading vaccine candidate and a prime target in irradiated sporozoite immunity^6,10–14^. CSP plays multiple essential functions throughout pre-erythrocytic stage development including motility, cell traversal, and liver stage development (reviewed in^15^). Recent studies have demonstrated that CSP-based vaccines can elicit significant protection after sporozoite challenge^16,17^ and attenuate liver-stage (LS) development^18^. This molecule forms the basis of the *Plasmodium falciparum* malaria vaccines (RTS,S/AS01 and R21/MM) currently authorized for use in children in endemic regions^19–21^. However, very limited progress has been achieved towards a *P. vivax* CSP based vaccine^22–29^. Other studies suggested protective anti-PvCSP memory B-cells are short lived due to poor immunogenicity^30,31^. Thus, there is a need to identify other potential candidates to partner with CSP in a multivalent vaccine to protect against infection and disease.

The cell-traversal protein for ookinetes and sporozoites (CelTOS) is another PE antigen, which is highly conserved among *Plasmodium* spp.^32^ and considered an attractive vaccine candidate. It plays a critical role in sporozoite egress from host cells during traversal^33–36^, which is a necessary part of ookinete infection of the mosquito midgut and sporozoite infection of the liver. *P. vivax* CelTOS is naturally immunogenic^37,38^ and immunization of mice with recombinant CelTOS elicits both humoral and cellular immune responses that reduced hepatocyte infection^34,37,39–52^. Antibodies targeting CelTOS can inhibit gliding motility, cell traversal, sporozoite hepatocyte infection, and impaired parasite development in the mosquito^39,52^. Parasites lacking expression of CelTOS are defective in mosquito and liver stage development^33^, suggesting its importance as both a transmission blocking and PE vaccine target.

In a recent study on transcriptional profiling of *P. vivax* sporozoites, we identified additional *P. vivax* sporozoite antigens including the sporozoite surface protein 3 (SSP3) and sporozoite protein essential for cell traversal (SPECT1), which are upregulated in response to changes in their microenvironment and are associated with host cell infectivity^53^. In the rodent malaria parasite *Plasmodium berghei*, SSP3 is predominantly located on the sporozoite surface and is shown to play a role in gliding motility and/or liver stage development. SPECT1 plays a role in pore formation, which is essential for traversal of the liver sinusoid and Küpffer cells^49,54–56^.

In this study, we evaluated the naturally acquired antibodies to *P. vivax* PE antigens CSP (VK210 allele), CelTOS, SSP3 and SPECT1 in plasma samples from *vivax* infected patients from two endemic regions in Thailand. At least 80% of the study subjects had antibodies to all four antigens and these antibodies inhibited sporozoite infection and hepatocytes development in vitro. Understanding the magnitude and quality of naturally acquired antibody responses to these sporozoite antigens in endemic populations will guide target selection for inclusion in a multi-valent PE vaccine aimed at generating protective antibodies against sporozoites infection and development in hepatocytes, preventing blood stage infection and disease.

## Results

### Naturally acquired antibodies to *P. vivax* PE antigens

**Figure 1:**
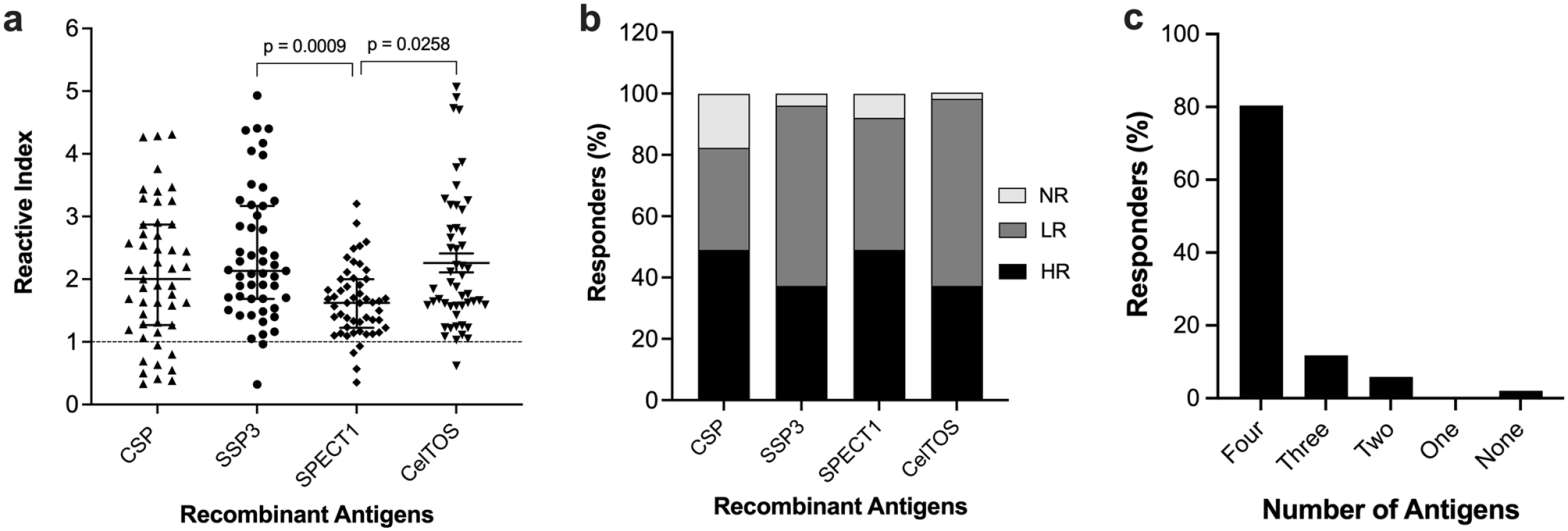
Profile of naturally acquired IgG antibodies against PE antigens. (**a**) Antigen-specific antibody levels in plasma samples from Thai *P. vivax* infected patients (n = 51) were measured by indirect ELISA against recombinant CSP, SSP3, SPECT1, and CelTOS. Data is expressed as reactive index (RI) units, calculated by dividing the mean OD value of each patient sample by a cut-off value. The cut-off value was calculated as the mean OD + 2 SD of 6 naïve North American control samples^75^. Each point on the graph represents individual RI units with median and interquartile range. Plasma samples with RI < 1 (dotted lines) are considered not reactive (**b**) Frequency of IgG antibodies against respective antigens classified into HR (RI ≥ mean reactivity of all samples), LR (RI < mean reactivity of all samples but > cut-off value), and NR (RI < cut-off value). (**c**) Number of individuals with cross-reactive IgG antibodies to the different antigens.

Plasma samples from 51 Thai patients with acute *P. vivax* infections were screened for the presence of antigen-specific antibodies to selected PE antigens including CSP, SSP3, SPECT1 and CelTOS. There was considerable variation in magnitude of IgG antibody responses in individual patient samples against the different antigens, with significant differences in overall response observed between SSP3, CelTOS and SPECT1 (Fig. 1a). Based on the reactivity profiles, antigen specific responses could be categorized into three response groups: high responders (HR), defined as samples with antibody reactivity index (RI) greater than the mean reactivity of all samples per antigen, low responders (LR), with RI less than the mean reactivity of all samples but greater than the cut-off value (RI = 1) and non-responders (NR) with RI less than or equal to the cut-off value (Fig. 1b). Out of the 51 samples screened, 25 (49%) of them were HR for CSP and 25 (49%) for SPECT1. Similarly, SSP3 and CelTOS had the same number of HR at 19 (37.3%). However, these were not the same samples for each antigen. A total of 17 (33.3%), 30 (58.8%), 22 (43.1%) and 31 (60.8%) of samples were LR for CSP, SSP3, SPECT1 and CelTOS, respectively. Independent of their responder classifications, about 80% plasma samples had antibodies to all four antigens, 12% to three antigens, 6% to two antigens and no sample had antibodies to a single antigen. Only 2% of samples did not have antibodies to any of the four antigens (Fig. 1c). The prevalence of antigen specific antibodies was 98% for CelTOS, 96.1% for SSP3, 92.2% for SPECT1 and 82.4% for CSP (Fig. 1c).

### Antibodies in plasma of Thai patients inhibit sporozoites invasion of hepatocytes

**Figure 2:**
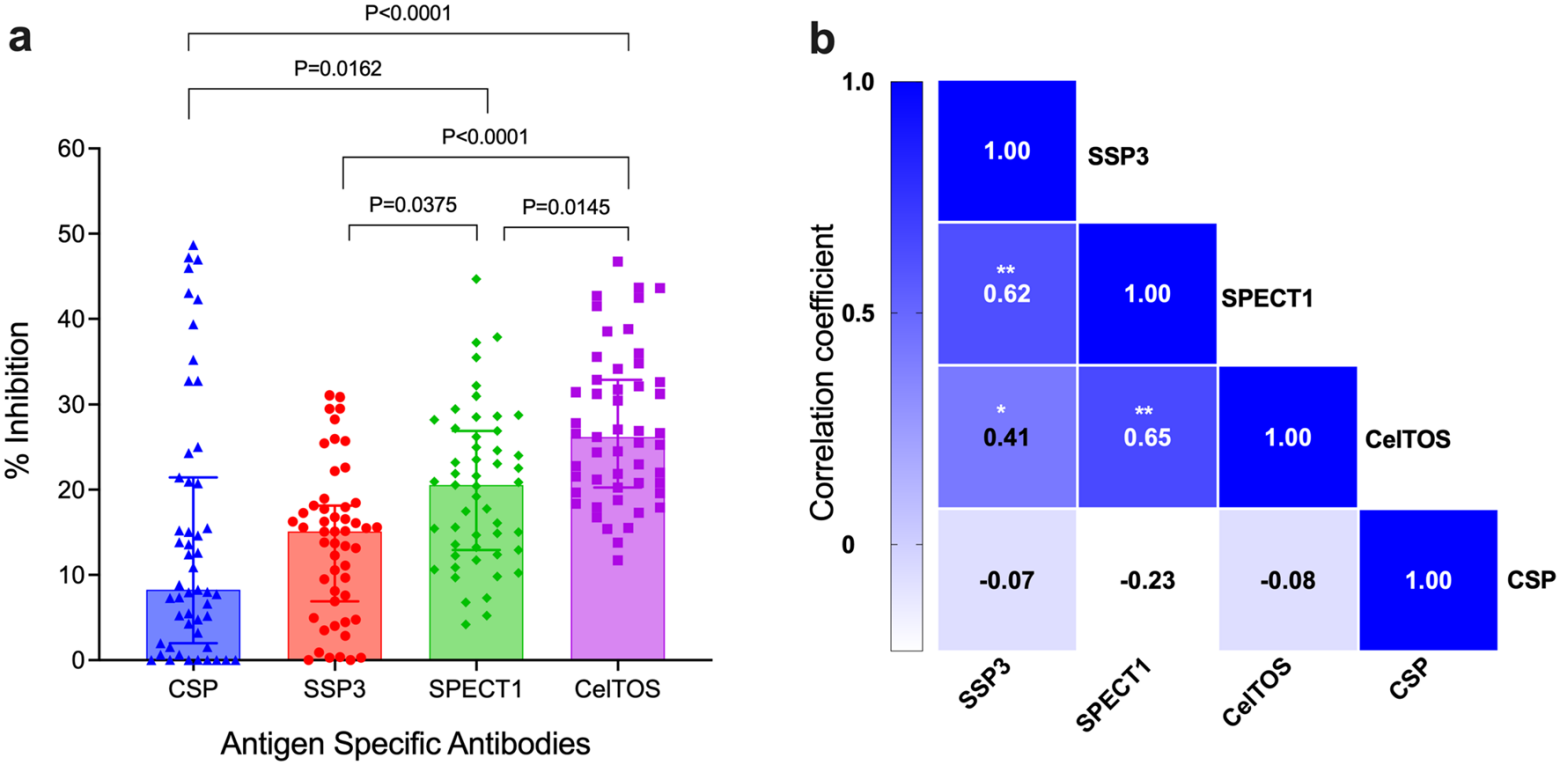
Inhibition of sporozoite infectivity of human hepatocytes in vitro. (**a**) Sporozoites of *P. berghei* transgenic parasites expressing *P. vivax* CSP, SSP3, SPECT1, CelTOS, were pre-exposed to plasma samples from vivax-infected patients (n = 51) before being added to a monolayer of HC-04 cell line in 384 well plates. Inhibition of hepatocyte infection was expressed as percent inhibition in the formation of EEFs in the presence of patient plasma (test samples) relative to the naïve North American control plasma. Each point on the graph represents the percent inhibition of individual plasma and bars represent the median and interquartile range for two independent experiments tested in triplicate. Statistical analysis was performed by Kruskal–Wallis test Dunn’s multiple comparison analysis. (**b**) Correlation of antibody inhibitory responses between *PE* antigens. Pairwise Spearman’s correlations were determined by percent inhibition in ILSDA between each antigen pair. Correlation was performed to predict inhibitory interactions between PE antigens. Antigen pairs with Spearman r > 0.6 and adjusted *p*-value < 0.0001 suggest a moderately significant correlation. Significance indicated by *p < 0.005, ** *p* < 0.0001.

The potential protective effect of naturally acquired antibodies to these antigens on sporozoite invasion of hepatocytes was evaluated by an in vitro inhibition of liver stage development assay (ILSDA). This assay is performed in 384-well plate, and makes use of a human hepatoma cell line (HC-04) and transgenic *P. berghei* sporozoites expressing the different *P. vivax* PE antigens to support high level liver infections and parasite development to blood stage breakthrough^18^. Antigen specific inhibitory antibodies were present in the patient samples and inhibited invasion of transgenic sporozoites into hepatocytes. There was a wide variation and significant differences in the levels of antigen specific inhibitory antibody responses of individual plasma samples**,** with antigen inhibition ranging from 0 to 50% at the tested plasma dilution of 1:100 (Fig. 2a). The highest inhibitory effect was observed with anti-CelTOS antibodies, with mean percent inhibition ranging from 11 to 46.6% (mean = 27.3%), followed by SPECT1 with 4–44.6% (mean = 20.2%), SSP3 with 0–31.1% (mean = 13.9%) and CSP with 0–48.6% (mean = 14.7%). Spearman pairwise correlation of antibody inhibition between antigens demonstrates a moderately significant correlation between anti-SSP3 and anti-SPECT1 antibodies (r = 0.62 and *p* < 0.0001) as well as anti-SPECT1 and anti-CelTOS antibodies (r = 0.65, *p* < 0.0001), while a relatively weaker correlation albeit statistically significant was found between anti-SSP3 and anti-CelTOS (r = 0.41, *p* = 0.003) (Fig. 2b). No correlation of inhibition was observed between anti-CSP antibodies and antibodies against SSP3, SPECT1 or CelTOS. There was no correlation between antibody titer (RI) and inhibition of sporozoite invasion into hepatocytes (Supplementary Fig. 1).

## Discussion

Characterization of naturally acquired antibody responses to *P. vivax* antigens following *vivax* infections is an important step towards target selection and rational vaccine design to protect against vivax malaria. Naturally acquired IgG antibody responses to *P. vivax* antigens has focused mainly on blood-stage antigens rather than pre-erythrocytic antigens^57,58^. *Plasmodium* sporozoites injected by the mosquito migrate to the liver where they infect hepatocytes and develop into liver-stage forms or exoerythrocytic forms (EEFs)^49,59^. Since sporozoite antigens play a critical functional role during this migration process, they are considered potential vaccine targets. To determine if PE antigens are naturally immunogenic and could induce protective antibody responses in residents of endemic regions, quantitative and qualitative analysis of IgG antibody responses to four *P. vivax* PE antigens: CSP, SSP3, SPECT1 and CelTOS were evaluated in 51 plasma samples from acute symptomatic vivax infected patients from a low endemic region of Southern Thailand. Antigen specific antibodies were prevalent in the study subjects, with considerable heterogeneity in magnitude of antibody responses (Fig. 1). Over 80% of the patients had antibodies to all four antigens, suggesting that epitopes of these antigens are immunogenic and commonly exposed to the host immune responses during natural infections. However, the lack of correlation between the antibodies’ titer and level of functional inhibition suggest epitope specificity and strain variation may be important variables in development of protective immune antibodies.

Anti-CelTOS specific antibodies had the highest frequency, with 98% seropositivity, followed by anti-SSP3, anti-SPECT1 and anti-CSP antibodies with 96%, 92% and 82% respectively. In a low endemic region in Western Thailand where a majority of the study population was not infected, CelTOS was not substantially immunogenic^57^, while in the Brazilian Amazon, only 17.8% (94/528) of study subjects had specific CelTOS IgG antibodies^37^. This later study demonstrated that high antibodies against CelTOS were driven by cytophilic antibodies, suggesting that antibody response to CelTOS might be associated with recent infections^37^, which could explain the high prevalence of anti-CelTOS antibodies in our study. However, Longley et al.^57^ demonstrated that anti-CelTOS antibodies could last up to 1 year in the absence of *P. vivax* infection.

We also demonstrated the prevalence of naturally acquired anti-CSP antibodies in our study subjects. Other studies also reported the prevalence of anti-CSP antibodies in other endemic regions, some of which could last between 5 and 12 months even in the absence of detectable exposure to *P. vivax* infection^57,60–62^, with most of the antibody response biased towards the immunodominant central repeat region. The other *P. vivax* PE stage antigens, SSP3 and SPECT1, are also known to play important roles in sporozoite migration and infectivity. Most data on SSP3 and SPECT1 are based on studies with the rodent malaria parasite *P. berghei*^55,56^. Currently, there are no published data on the acquisition of naturally acquired immunity to *P. vivax* SSP3 and SPECT1 in endemic regions. Here, we showed that these two antigens are naturally immunogenic, with SSP3 showing significantly higher antibody responses than SPECT1 in the study subjects (Fig. 1).

The association between patient age and the presence of antibodies to these PE antigens was evaluated to determine if there was a correlation between the acquisition of naturally acquired immunity to malaria and age. The Median (interquartile range; IQR) age of participants was 32 (20–44) years old. No correlations were found between antibody levels to these antigens and the age of study participants (Supplementary Fig. 2), although future studies with a larger study population will be needed to validate this finding. Nonetheless, these data mirror studies with blood-stage antigen PvAMA-1^63^ and liver-stage antigen, PvTRAP^64^, which demonstrated no correlations between age and antibody levels in individuals with acute symptomatic *P. vivax* infection.

Orthologues of these PE stage antigens have been shown to play important roles in gliding, traversal, and invasion in other *Plasmodium* species^49,54,65–67^. Antibodies to PfCelTOS inhibited sporozoite motility and hepatocyte invasion in mice immunizations and sterile immunity in a heterologous *P. berghei* sporozoite challenge^39,41^. Anti-PfCSP human antibodies are also associated with protective immunity^68^. In *P. vivax,* monoclonal antibodies to CSP not only block sporozoite entry but also could inhibit subsequent development within the hepatocytes in vitro as evidenced by abnormal EEFs^69^. To determine the potential vaccine efficacy of antibodies to the *P. vivax* PE antigens, we evaluated naturally acquired antibodies in patient samples for inhibition of sporozoites invasion of human hepatocytes by ILSDA. This assay platform has been validated for evaluating inhibition of liver stage developmental and to assess the specificity and sensitivity of both *P. falciparum* and *P. vivax* antibodies^6,18,70^. We showed that naturally acquired antibodies to these PE antigens in patient samples inhibited transgenic *P. berghei* sporozoites expressing the different PE stage antigens from invading hepatocytes in vitro (Fig. 2), suggesting the presence of functional epitopes of naturally acquired protective antibodies on these antigens. Overall, the highest inhibitory responses were against CelTOS, followed by SPECT1. Although a few individuals had high inhibitory antibodies against CSP and SSP3, most of them had either very low or no inhibitory antibodies at all. It should be noted that of the three naturally occurring *P. vivax* CSP variants, which differ in the immunodominant repeat regions, only the CSP-VK210 variant was assessed in this study, which might account for the high rate of non-responders and low or non-inhibitory antibodies against CSP if the repeat regions are the primary targets of inhibitory antibodies. Thus, there is a possibility that some of the observed non-responders and non-inhibitory samples of CSP could be responders and inhibitory against a different CSP variant. In the case of SSP3, it could be possible that the use of a truncated SSP3 protein (amino acids 19-203) may have resulted in a low estimate for the SSP3 titers.

Interestingly, a sample with high inhibitory antibodies (% inhibition > 30%), against one antigen did not necessarily show high inhibition against the other antigens. Only 1 individual had high inhibitory antibodies against all three antigens (CelTOS, SPECT1, SSP3), 2 individuals against two antigens (CelTOS, SPECT1 or SSP3) and no individual with inhibitory antibodies against all four antigens. There was no correlation between high titer anti-CSP inhibitory antibodies and inhibitory antibodies to any of the other three antigens. Although other parasite factors such as the specific strain of the parasite and host immune factors including previous exposure play a role in the development of immunity against these different antigens, our data suggest that the acquisition of functional antibodies against each antigen is independent of the other and support the need for identifying potential PE antigens to partner with CSP in a multivalent vaccine targeting different PE antigens. Thus, identifying parasite antigens that are targets of naturally acquired inhibitory antibodies are ideal candidates for inclusion in such a vaccine to prevent hepatocyte infection and the onset of clinical disease by blocking the progression of the parasite in the liver to blood-stage breakthrough.

In summary, we showed that naturally acquired antibodies to the selected *P. vivax* PE antigens were prevalent in the study subjects from Thailand, but individuals had significant quantitative and qualitative differences in their antigen-specific antibody responses. Secondly, we demonstrated that there was no correlation between antibody titer (defined by RI), age of patients, and functional inhibitory activity against each antigen. This finding mirrors results previously reported for the Duffy binding protein (DBPII), which is a leading *P. vivax* blood-stage vaccine candidate^71^. This study is the first to investigate the functional activity of naturally acquired antibodies to these four *P. vivax* PE antigens. Together, our data highlights the potential of these PE antigens as vaccine targets and supports their inclusion in vaccine designs aimed at targeting sporozoite invasion of hepatocytes and liver stage development. Preventing parasites from developing in the liver and progressing to the blood stage will not only prevent the development of clinical disease and morbidity but also prevent transmission. Additional studies are needed to investigate the dynamics involved in the acquisition of antigen-specific antibodies and the frequency of vivax infections in the study subjects. Further studies are required to assess the magnitude, longevity and factors affecting the acquisition of antigen-specific antibodies with a larger cohort of patients.

## Materials and methods

### Ethics statement

This study was approved by the Committee on Human Rights Related to Human Experimentation, Mahidol University Central Institutional Review Board (MU-IRB 2012/079.2408 and MU-CIRB 2021/281.2505), and the Committee on Use of Human Subjects in Research, University of South Florida Institutional Review Board (IRB-Pro00018143). All the study participants or their legal guardians provided written informed consent.

### Study population and sample collection

A total of 51 plasma samples were obtained from acute symptomatic *P. vivax-infected* patients in an area of low transmission in the provinces of Ranong (n = 23) and Chumphon (n = 28) in southern Thailand. In these regions, both *P. vivax* and *P. falciparum* infections are common, with an average of 40 vivax malaria cases per year based on 2017–2022 data from the Thai Department of Disease Control, Ministry of Public Health. Samples were collected during the rainy season, between August and December, which coincides with the high transmission season. Self-reporting study participants were screened for vivax malaria by microscopy of Giemsa-stained smears and confirmed by PCR. Only individuals aged 18 and above, and positive for *P. vivax* infection were recruited for this study. Other inclusion criteria included a systolic blood pressure greater than 90 mmHg, body temperature less than 40 °C, and hematocrit greater than 25%. Blood samples were collected from patients in heparinized tubes and the plasma was separated and stored frozen until needed. Plasma samples from six naïve North American volunteers were used as controls.

### Recombinant protein production

Recombinant proteins were produced from four *P. vivax* pre-erythrocytic (PE) vaccine candidates: SPECT1 (PVP01_1212300), SSP3 (PVX_123155), CelTOS (Sal1, PVX_123510) and CSP (PVP01_0835600.1). For CSP and SPECT1 the signal peptides and transmembrane domains were excluded in expressed recombinant proteins, while for SSP3, only the N-terminal region from residues 19-203 was expressed^72^. The gene coding for CSP was codon optimized for *Escherichia coli* expression and cloned into pET21(a +) expression vector with a C-terminal hexahistidine tag. Recombinant CSP was expressed in *E. coli* BL21 Star (DE3) strain and purified on HisTrap HP by affinity chromatography using the Akta Pure System (GE). The production of rSSP3 and rSPECT1 was previously reported^72^. Recombinant CelTOS was expressed in *E. coli*, purified by Nickel-NTA chromatography and gel filtration as previously reported^34,35,73,74^. The purity of recombinant proteins was evaluated by SDS-PAGE.

### Assessment of antibody response to *P. vivax* PE antigens

Plasma samples from vivax-infected patients (n = 51) were screened for antibody responses to recombinant *P. vivax* antigens CSP, CelTOS, SSP3, and SPECT1 by indirect ELISA. Briefly, 96-well microtiter plates were coated overnight at 4 °C with 100 µl/well of recombinant proteins at 3 µg/ml in PBS. Coated plates were washed with PBS/0.05% Tween-20 (PBS-T) and blocked with 5% (w/v) skimmed milk in PBS-T for 2 h at room temperature. Plasma samples diluted 1:200 with 2% milk in PBS-T were added to duplicate wells and incubated on a shaker for 2 h at room temperature. Six naïve North American plasma samples were tested on each plate as negative controls, and wells without coated antigens were used for background control. After another PBS-T wash, wells were incubated with a phosphatase-labelled goat anti-human IgG (H + L) antibody (SeraCare) for 90 min at room temperature. Bound antibodies were detected after development with 100 μl of phosphatase substrate solution (SeraCare) and optical density (OD) values were measured at 650 nm. Antibody responses were reported as Reactive Index (RI)^75^. RI was calculated by dividing the OD of test sample by a cut-off value determined for each plate. Cut-off value = Mean OD values + 2 SD of 6 naïve North American control samples. RI = (OD_TS_–OD_BK_)/(Mean OD_CS_ + 2 SD), where TS = test sample, BK = blank, CS = control samples, and SD = standard deviation. A sample with RI ≥ 1 (cut-off value) was considered a responder, while RI < 1 is considered a non-responder.

### Animal studies

Female Swiss webster ND4 mice (6–8 weeks; Envigo, USA) were used in the current study and all animal procedures were performed in compliance with relevant guidelines and regulations in IACUC protocol IS00008179 approved by the Division of Research Integrity and Compliance, University of South Florida. Mice were housed in ventilated cages and maintained at 21 °C with 12:12 h light–dark cycles at a relative humidity of 55 ± 10%. All animal studies are reported following ARRIVE guidelines^76^.

### Parasites

The following *P*. *berghei* ANKA–*P. vivax* transgenic parasite lines were used: (i) 2321cl3, *Pb-Pv*CelTOS(r) where endogenous *P. berghei* CelTOS is replaced with *P. vivax* CelTOS (RMgm-4111, www.pberghei.eu)^50^. (ii) 3378cl1, *Pb-Pv*SPECT1(r) where endogenous *P. berghei* SPECT1 is replaced with *P. vivax* SPECT1, (iii) 3392cl1, *Pb-Pv*SSP3(r) where endogenous *P. berghei* SSP3 is replaced with *P. vivax* SSP3 and (iv) Pb05cl1, *Pb-Pv*CSP P01(r) where endogenous *P. berghei* CSP is replaced with *P. vivax* CSP from P01 reference strain (Kolli SK, et al., manuscript in preparation). *Pb-Pv*CelTOS(r) and *Pb-Pv*SPECT1(r) transgenic lines express *gfp-luciferase* fusion reporter gene under the control of constitutive *Pbeef1α* promoter integrated into the neutral *230p* gene locus^77^. *Pb-Pv*SSP3(r) and *Pb-Pv*CSP P01(r) express mCherry and luciferase reporter genes under the constitutive *Pbhsp70* and *Pbeef1α* promoters, respectively integrated into the neutral *230p* gene locus^78^.

### Cell lines

Human hepatocyte cell line HC-04 (MRA-975) obtained from BEI resources were cultured in Minimum essential medium (Gibco) and Ham’s F12 nutrient mix (Gibco) supplemented with 10% heat-inactivated fetal bovine serum (GenClone), 30 mM HEPES (Gibco), 2 mM L-Glutamine (Gibco) and 40 µg/ml Gentamicin (Sigma-Aldrich) and maintained at 37 °C with 5% CO2 in a collagen coated flask (Corning). The cell line was tested negative for mycoplasma contamination (Invitrogen) and authenticated via American Type Culture Collection Human STR Profiling Service (ATCC).

### Production of transgenic *P. berghei* sporozoites

Groups of female Swiss Webster ND4 mice (n = 2), were infected with a cryopreserved stock of respective *P. berghei* transgenic parasites line intraperitoneally. At 2–5% parasitemia and comparable gametocytemia, three to five days old female *Anopheles stephensi* mosquitoes were allowed to feed on the mice that were under anesthesia for 15–20 min. The mice were then euthanized with CO_2_ after mosquitos’ infection. Infected mosquitoes were maintained at 21.5 °C and 80% relative humidity and supplied with 5% glucose ad libitum. Infectivity of mosquitoes was assessed by counting the number of oocysts on day 14 post feeding. Sporozoites were isolated by manual dissection of mosquito salivary glands on day 18–21 post feeding and collected in Leibovitz’s L-15 medium (Thermos Fisher Scientific). The glands were centrifuged at 6000 rpm for 1 min and mechanically disrupted using a plastic pestle to release the sporozoites. The crushed sporozoite solution was filtered through a 40 µM cell strainer (Greiner Bio-One) and sporozoites were counted using a hemocytometer.

### Inhibition of liver stage development assay (ILSDA)

Collagen coated 384 well plate (Greiner Bio-One) was seeded with HC-04 cells at a density of 8000 cells/well for 16 h before infecting with transgenic *P. berghei* sporozoites. Sporozoites (75 spz/µl) were incubated in 1:100 dilution of patient plasma or North American naïve plasma for 20 min at room temperature. After incubation, 3 × 10^3^ sporozoites per well were added to triplicate wells. The plate was centrifuged at 200 × g for 5 min and incubated at 37 °C in CO_2_ incubator to allow hepatocyte invasion. After 1 h, culture media was changed to remove uninvaded sporozoites and a subsequent media change 24 hpi. At 48 hpi, EEFs of transgenic lines expressing mCherry were imaged using live fluorescence and the nuclei stained with Hoechst 33342 for 30 min at 37 °C. Images were acquired using high content imaging system, Cell Insight CX7, at 20X objective.

The EEFs of transgenic lines expressing GFP were fixed with 4% PFA and EEFs were imaged after performing an indirect immunofluorescence assay as previously described with minor changes^63^. Briefly, the PFA from the wells was washed off twice with PBS and the parasites stained by incubation with goat polyclonal *P. berghei* UIS4 antibody (LS Bio) at 1:1000 dilution in blocking buffer (1% BSA and 0.3% Triton X-100) at 4 °C overnight. The wells were washed twice with PBS and counter stained with an anti-goat Alexa Fluor 594 conjugated secondary antibody (Invitrogen) and Hoechst 33342 (Invitrogen) to stain the nuclei for 1 h at 37 °C. The wells were again washed with PBS and incubated in fresh PBS. Images were acquired using high content imaging system, Cell Insight CX7, at 20X objective. The images were exported, and parasites counted using an in-house Python program (Supplementary Methods Text S1).

Live fluorescence imaging of mCherry expressing EEFs were counted using parasite cytoplasmic (PCP) staining whereas GFP expressing parasites were counted using UIS4 positive staining of parasitophorous vacuolar membrane (PVM), and Hoechst 33342 for host nuclei staining. Two independent assays were performed for each serum.

% inhibition of infection = 100–(Mean EEFs in test sample divided by Mean EEFs in 6 naïve North American samples) × 100.

### Statistical analysis

All data were tested for normality using the Anderson–Darling test before analysis. Statistical differences for ELISA data were determined using the Kruskal–Wallis non-parametric test with a Dunn’s multiple comparison adjustment. Statistical differences for sporozoite invasion inhibition were also determined with the Kruskal–Wallis non-parametric test with a Dunn’s multiple comparison adjustment. A Spearman Correlation was done on the overall percent inhibition for each antigen. Analysis was performed using GraphPad Prism v. 10.0.2 for MacOS.

### Supplementary Information

Supplementary Information.

## Data Availability

The datasets generated during and/or analyzed during the current study are available from the corresponding author on reasonable request.
