# Supplementary material for: Naturally acquired antibodies against Plasmodium vivax pre-erythrocytic stage vaccine antigens inhibit sporozoite invasion of human hepatocytes in vitro
Source: Sci Rep. 2024 Jan 13;14:1260. doi: 10.1038/s41598-024-51820-2 (PMC10787766; doi:10.1038/s41598-024-51820-2)
Supplement: Supplementary file 1 — Supplementary Information. [file 41598_2024_51820_MOESM1_ESM.docx]

**Naturally acquired antibodies against *Plasmodium vivax* pre-erythrocytic stage vaccine antigens inhibit sporozoite invasion of human hepatocytes *in vitro*.**

**Francis Babila Ntumngia^1*†^, Surendra Kumar Kolli ^1†^, Pradeep Annamalai Subramani^1†^, Samantha J. Barnes^1†^, Justin Nicholas^1,2^, Madison M. Ogbondah^1^, Brian B. Barnes^3^, Nichole D. Salinas^4^, Pongsakorn Thawornpan^5^, Niraj H. Tolia^4^, Patchanee Chootong^5^, John H. Adams^1*^**

^1^Center for Global Health and Interdisciplinary Research, University of South Florida, Tampa, FL, USA

^2^Department of Molecular Medicine, Morsani College of Medicine, University of South Florida, Tampa, FL, USA

^3^ College of Marine Science, University of South Florida, St Petersburg, FL, USA

^4^Host Pathogen Interactions and Structural Vaccinology Section, Laboratory of Malaria Immunology and Vaccinology, National Institute of Allergy and Infectious Diseases, National Institutes of Health, Bethesda, MD, USA

^5^Department of Clinical Microbiology and Applied Technology, Faculty of Medical Technology, Mahidol University, Bangkok, Thailand

^†^ Authors contributed equally to this work

**Running Title:** Naturally acquired antibodies to *P. vivax* Pre-erythrocytic stage antigens

***Correspondence:**

John H. Adams, PhD. Email: [ja2@usf.edu](mailto:ja2@usf.edu)

Francis B. Ntumngia, PhD. Email: fntumngi@usf.edu

**Key Words:** Malaria, naturally acquired immunity, *Plasmodium vivax*, pre-erythrocytic stage, vaccine, CSP, CelTOS, SSP3, SPECT1

**Supplementary Methods Text S1**

**Python code and image processing**

Three different channels were used for image acquisition: mCherry expressing EEFs via the parasite cytoplasmic (PCP) staining, GFP expressing parasites from UIS4 positive staining of parasitophorous vacuolar membranes (PVM), and Hoechst 33342 for host nuclei staining. To enumerate the parasites, a Python code was developed which incorporates image processing techniques and threshold-based identification of both host nuclei and parasites. For each image channel, the greyscale (byte; 0-255) image data were first processed using a Gaussian filter (kernel width = 2) to blur edges and remove background noise. Any pixels above a ‘background’ threshold (identified via trial-and-error as 10) were then identified, and a distance matrix was used to assess distance from each such identified pixel to the nearest non-identified pixel. Using an 11 x 11-pixel kernel, regional maxima in this distance matrix were identified and enumerated. The watershed transform was then applied on the distance matrix to segment the channel, after which any pixels below the background threshold were again removed. Each identified feature (likely a parasite or nucleus, depending on the stain) was then compared to pre-determined size thresholds (area of 122 to 24,000 pixels, which corresponds to radius of 5 to 70 μm). Any features outside of these bounds were excluded.

Results from these individually-processed channels were then merged. First, all pixels identified in both the PCP and PVM channel results were located. From this binary classification, a new distance matric was developed, followed by regional maxima determination (kernel size of 21) and watershed segmentation. Any such identified parasite feature whose mean PVM value was deemed too large (> 100) was removed as likely artifact. Similarly, any identified parasite feature that was farther than 1 μm from the nearest identified nucleus was removed. Finally, any features outside the size bounds listed above were removed, yielding the final parasite identification.

For each image, the size and location of all identified parasites was output in .png format, with dimensions equal to that of the input imagery. Additionally, the location of each parasite feature that had been excluded during the image processing (i.e., according to the size, brightness, or nuclei proximity logic) was also noted in these output images to highlight boundary cases, allowing easy assessment of the exclusion thresholds used. This output was manually reviewed to ensure accuracy of the image processing resul

**Supplementary Figures**

**Supplementary Figure S1.** Correlation between IgG antibody titer and inhibition of infectivity. Antibody titers were determined by indirect ELISA and functional activity determined using the inhibition of liver stage development assay (ILSDA). Pairwise Spearman’s correlations were determined between RI units and percent ILSD for each antigen. Coefficients of determination (*r*), Spearmen’s (*p)* value are shown.


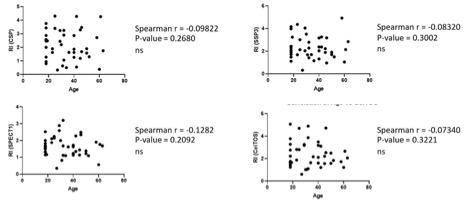


**Supplementary Figure S2.** Correlation analysis between IgG antibody titer (RI) and age of patients. Antibody titers were determined by indirect ELISA and OD values converted to Reactive Index (RI) as described in materials and methods. Pairwise Spearman’s correlations were determined between RI units and age of patients for each antigen. Coefficients of correlation (*r*) and Spearmen’s (*p)* values are shown.
